# *Alpinia officinarum* Hance: a comprehensive review of traditional uses, phytochemistry, pharmacokinetic and pharmacology

**DOI:** 10.3389/fphar.2024.1414635

**Published:** 2024-08-16

**Authors:** Xia Lei, Jiapeng Wang, Kun Zuo, Tianli Xia, Jinfeng Zhang, Xiangyue Xu, Qing Liu, Xiaoliang Li

**Affiliations:** ^1^ Jiangsu MC Clinical Innovation Center of Degenerative Bone and Joint Disease, Wuxi TCM Hospital Affiliated to Nanjing University of Chinese Medicine, Wuxi, China; ^2^ Engineering Research Center of Tropical Medicine Innovation and Transformation of Ministry of Education and International Joint Research Center of Human-Machine Intelligent Collaborative for Tumor Precision Diagnosis and Treatment of Hainan Province and Hainan Provincial Key Laboratory of Research and Development on Tropical Herbs and Haikou Key Laboratory of Li Nationality Medicine, School of Pharmacy, Hainan Medical University, Haikou, Hainan, China; ^3^ Hubei University of Chinese Medicine Affiliated Gongan Hospital of Traditional Chinese Medicine, Wuhan, China

**Keywords:** *Alpinia officinarum* Hance, traditional uses, phytochemistry, pharmacology, pharmacokinetic

## Abstract

The dried root and rhizome of *Alpinia officinarum* Hance (*A. officinarum*) have been widely used in traditional Chinese medicine for thousands of years to alleviate pain, promote digestion, warm the stomach, and disperse cold. This review aims to comprehensively and in-depth summarize the most recent research on the traditional uses, phytochemistry, pharmacokinetics, and pharmacology of *A. officinarum*. By searching various databases including Web of Science, PubMed, Google Scholar, Elsevier, Springer, ScienceDirect, and China National Knowledge Infrastructure (CNKI) for literature on “*A. officinarum* Hance,” as well as relevant textbooks and digital documents, an overall and critical review of the subject was conducted. The traditional uses of *A. officinarum* were summarized, and 337 compounds from *A. officinarum* were summarized, including flavonoids, diarylheptanoids, volatile oils, and other compounds. Studies have found that the crude extract of *A. officinarum* and its compounds has a wide range of biological activities, such as improving gastrointestinal function, anti-inflammatory properties, anti-tumor activity, antibacterial properties, memory enhancement, and analgesic effects. Modern pharmacological studies have provided strong evidence and explanations for the traditional medicinal uses of *A. officinarum*, which brings a broad prospect for its medicinal use. However, more research is needed to explore the structure-activity relationship and potential mechanisms of action of its bioactive chemicals. Furthermore, it is essential to conduct more clinical trials in order to accelerate research and development of the drug.

## 1 Introduction

With the development of the times, people are increasingly focusing on their wellbeing. The advancement of medical technology has also begun to attract attention. While new drugs for various diseases are constantly being developed, people are actively exploring alternative therapies and natural products due to the toxic side effects of chemical drugs and the uncontrollable risks of biological agents. *Alpinia officinarum* Hance (*A. officinarum*), native to China, is one of the most important species of the Zingiberaceae family, which is widely distributed in Fujian, Taiwan, Guangdong, Guangxi, Hainan, and other provinces in China (Sun et al., 2023; Zheng et al., 2024). The detailed description of the medical applications of *A. officinarum* can be traced back to the book “Ming Yi Bie Lu,” which was written during the Han Dynasty (Tao, 1986). As a medicinal part, the aromatic rhizome of *A. officinarum* mainly belongs to the spleen and stomach meridians and was widely used in the treatment of gastrointestinal diseases in ancient China (Tushar et al., 2010; Al Garni et al., 2024).

Botanical drugs have been widely used to treat many diseases for centuries due to their obvious effectiveness, fewer side effects, and relatively low cost. *A. officinarum* is known for its extensive clinical applications because it contains a variety of bioactive substances, including flavonoids, diarylheptanoids, volatile oils, phenylpropanoids, and glycosides (Pillai et al., 2018; Wen et al., 2024). Flavonoids and diarylheptanoids are its main components and have been proven to have a variety of pharmacological effects (Abubakar et al., 2018). In this paper, the traditional uses, chemical components, and biological activities of *A. officinarum* were reviewed comprehensively, which provide better guidance for the rational utilization of it.

## 2 Traditional efficacy and application of *A. officinarum*

*A. officinarum*, which is also known as “Liangjiang” and “Xiaoliangjiang,” was first recorded in the “Ming Yi Bie Lu” during the Han Dynasty (Tao, 1986). As shown in Table 1, the properties of *A. officinarum* have mainly been described as pungent and warm, while in some ancient books, there have been occasional records of “bitter”. It has been recorded in ancient books that *A. officinarum* mainly enters the two meridians of the spleen and stomach, but rarely enters the heart, liver and Danzhong meridians. The records of *A. officinarum* in modern works on herbal all belong to the spleen and stomach meridians. Through the analysis of the records of the efficacy of *A. officinarum* in ancient and modern Chinese botanical drug, it was found that its common features in terms of efficacy are warming the stomach, dispelling cold, relieving pain, regulating qi, stopping vomiting, and alleviating diarrhea. And, *A. officinarum* is commonly used to treat epigastric cold pain, vomiting, diarrhea, and food stagnation.

**TABLE 1:** Medicinal properties, meridian tropism, and efficacy of *A. officinarum*.

Dynasty	Book title	Author	Property and taste	Meridian tropism	Efficacy and application	Reference
Han Dynasty	Ming Yi Bie Lu	Hongjing Tao	Hot		Violent cold, coldness in the stomach, abdominal pain caused by cholera	Tao (1986)
Northern and Southern Dynasties	Ben Cao Jin Ji Zhu	Hongjing Tao	Hot		The same as the record of “Ming Yi Bie Lu”	Tao (1994)
Sui and Tang Dynasties	Xin Xiu Ben Cao	Jing Su	Hot		The same as the record of “Ming Yi Bie Lu”	Su (1981)
	Ben Cao Shi Yi	Zangqi Chen	Pungent, warm	Spleen and stomach meridians	Exsufflation, dysentery and cholera	Chen (1983)
Song, Jin and Yuan Dynasties	Kai Bao Ben Cao	Han Liu, Zhi Ma	Hot		The same as the record of “Ming Yi Bie Lu”	Liu and Ma (1998)
Ming Dynasty	Classified materia medica	Shenwei Tang	Hot		The same as the record of “Ming Yi Bie Lu”	Tang (1982)
	Tang Ye Ben Cao	Haogu Wang	Pungent, hot		Coldness in the stomach, abdominal pain caused by cholera, nausea, diarrhea, exsufflation and digestion	Wang (1998)
	Dian Nan Ben Cao	Mao Lan	Pungent, warm	Spleen and stomach meridians	Stomachache caused by qi or cold	Lan (1975)
	Ben Cao Meng Quan	Jiamo Chen	Pungent, bitter, hot		Invigorating spleen to promote digestion, cholera, diarrhea, nausea, coldness and pain of the abdomen	Zhang (1998)
	Compendium of materia medica	Shizhen Li	Pungent, hot	Spleen and stomach meridians	Invigorating the spleen and stomach, relieving dysphagia, breaking cold addiction, malaria	Li (2008)
	Ben Cao Hui Yan	Zhumo Ni	Pungent, hot	Spleen and stomach meridians	Dispelling cold and dampness, warming spleen and stomach	Ni (2015)
	Jing Yue Quan Shu	Jingyue Zhang	Pungent, hot	Spleen and stomach meridians	Stomach cold, vomiting, cholera, abdominal pain, antialcoholic	Zhang (2006)
	Ben Cao Tong Xuan	Zhongzi Li	Pungent, warm	Spleen and stomach meridians	Stop vomiting, diarrhea, eliminating malaria, elimination of overeating	Li (2015)
Qing Dynasty	Ben Cao Yi Du	Renan Wang	Pungent, bitter, hot	Spleen and stomach meridians	Promoting digestion, invigorating the spleen, Cold abdominal pain, vomiting	Wang (1987)
	Ben Jing Feng Yuan	Lu Zhang	Pungent, hot	Spleen and stomach meridians	Warming the spleen and stomach, stomach cold, cholera, abdominal pain	Zhang (2007)
	Ben Cao Ze Yao Gang Mu	Jiefan Jiang	Pungent, hot	Spleen and stomach meridians	Cold reflux in the stomach, cholera, abdominal pain	Jiang (1985)
	Ben Cao Xin Bian	Shiduo Chen	Pungent, hot	Heart, dan zhong, spleen and stomach meridians	Invigorating the spleen and stomach, stomach cold, diarrhea, abdominal pain	Chen (2008)
	Yu Qiu Yao Jie	Yuanyu Huang	Pungent, warm	Spleen and stomach meridians	Cold dampness of spleen and stomach, vomiting, cholera, malaria, dysentery, choking, malaria	Huang (2017)
	Ben Cao Cong Xin	Yiluo Wu	Pungent, hot		Warm the stomach and dissipate cold, cold pain in stomach duct	Wu (2001)
	De Pei Ben Cao	Jie Yan	Pungent, hot	Spleen and stomach meridians	Cold pain in stomach duct, cholera, diarrheum, malaria	Yan (1997)
	Ben Cao Qiu Zhen	Gongxiu Huang	Pungent, hot	Stomach meridian	Warming the stomach and eliminating food, treating cholera and diarrhea, vomiting and malaria	Huang (2012)
	Ben Cao Hai Li	Huan Ling	Pungent, warm, hot	Spleen, stomach, and liver meridians	Warming the stomach to remove choking diaphragm, heartache, malaria	Ling (1982)
	Ben Cao Bian Du	Bingcheng Zhang	Pungent, warm	Spleen and stomach meridians	Cold pain in the stomach and vomiting	Zhang (1958)
Modern	Chinese pharmacopoeia 2015	National pharmacopoeia committee	Pungent, hot	Spleen and stomach meridians	Cold abdominal pain, stomach cold vomiting, belching acid	Chinese Pharmacopoeia Commission (2015)
	Chinese materia medica	Liren Song, Yigu Wu, lie Hu	Pungent, hot	Spleen and stomach meridians	Cold abdominal pain, vomiting, belching	Song (1999)
	Great dictionary of chinese medicine	Mingsan Miao, Yuxin Sun, Xiaotian Wang	Pungent, warm	Spleen and stomach meridians	Cold spleen and stomach, cold abdominal pain, vomiting, diarrhea, food stagnation, malaria	JiangsuNewMedicalCollege (1999)
	National compendium of chinese herbs	Zongwan Xie, Cuisheng Fan, Zhaoyi Zhu	Pungent, warm		Cold stomach pain, acute gastroenteritis, sweat stain	CGONCHM. Compilation (1996)
	Zhong Yao Zhi	Pharmaceutical institute of the academy of medical science of china	Pungent, warm		Cold spleen and stomach, chest and abdomen pain, vomiting, choking, dyspepsia, diarrhea	Sciences (1959)

*A. officinarum* has been widely used in clinics due to its compatibility in many prescriptions, as shown in Table 2. *A. officinarum* is mainly used to warm the spleen and stomach, such as in Er Jiang Pill (Liu, 2017), which can nourish the spleen and stomach, remove cold, and eliminate phlegm, and cure all injuries caused by cold. Such prescriptions also include Wenzhong Liangjiang Pill (Liu, 2017) and Qing Zao San (Zhu, 2003). *A. officinarum* is a pungent and hot substance that is a pure yang product. It enters the spleen and stomach meridians, which can warm the stomach, reduce reflux and stop vomiting, and strengthen the spleen and stop diarrhea. For example, Ding Qi San (Zhao, 2018) is suitable for vomiting induced by typhoid. This type of prescription also includes Bi Cheng Qie San (Dou, 2015). *A. officinarum* can also enter the heart and Dan zhong, so it can enter the heart and pericardial meridian to warm and circulate qi. The prescriptions suitable for these kinds of conditions are Liang Fu Pill (Xie, 1990) and Gao Liang Jiang Decoction (Sun, 1955). With its fragrant and warm properties, *A. officinarum* can dissipate the cold, relieve pain, and promote qi. For example, Tian Tai Wu Yao San (Li, 1959) is applicable to the syndrome of cold coagulation and qi stagnation in the liver meridian. *A. officinarum* also has the effect of dispelling wind and relieving pain. The Qun Xun San, composed of *A. officinarum* and scorpion, has significant therapeutic effects on wind-induced toothache and swelling and pain in the cheek (Wang, 2003). In addition, *A. officinarum* has certain effects of warming the kidney and enhancing Yang. *A. officinarum* is compatible with *Tetradium ruticarpum* (A. Juss.) T. G. Hartley, which can warm the kidneys and dispel cold, and treat kidney deficiencies and waist pain. This type of prescription also includes Baji Pill (Liu, 2017).

**TABLE 2:** Prescription name, composition and therapeutic application of *A. officinarum*.

Book title	Prescription name	Composition	Therapeutic application	Reference
Tai Ping Hui Min He Ji Ju Fang	Er Jiang Pill	*A. officinarum*, Zingiber oj-jicinale Rosc	Nourish the spleen and warming the stomach, removing cold and eliminating phlegm, treating the pain of heart and spleen, and all injuries caused by cold	Liu (2017)
	Wen Zhong Liang Jiang Pill	*A. officinarum*, Rhizoma Zingiberis Preparata, *Atractylodes macrocephala* Koidz., *Cinnamomum cassia* (L.) D. Don, Glycyrrhizae Praeparata cum Melle Radix et Rhizoma	Cold phlegm gathering, Qi stagnation, vomiting after eating, vomiting, cold diarrhea, colic and tingling lateral thorax	
	Ba Ji Pill	*A. officinarum*, *Kadsura longipedunculata* Finet and Gagnep., *Morinda officinalis* F. C. How, *Cinnamomum cassia* (L.) D. Don, *Tetradium ruticarpum* (A. Juss.) T. G. Hartley	Deficiency of Yuan Qi, heavy waist and crotch, night sweat, chronic uterine coldness, irregular menses, leucorrhea, leukorrhea with bloody discharge	
Zhu Shi Ji Yan Fang	Qing Zao San	*A. officinarum*, Zingiber oj-jicinale Rosc, *Citrus reticulata Blanco*, *Glycyrrhiza uralensis* Fisch	Diarrhea, swelling and pain in the chest and abdomen	Zhu (2003)
Sheng Ji Zong Lu	Ding Qi San	*A. officinarum*, *Alpinia katsumadai* Hayata, Aucklandia lappa Decne., *Glycyrrhiza uralensis* Fisch	Vomiting during typhoid fever	Zhao (2018)
Bian Que Xin Shu	Bi Cheng Qie San	*A. officinarum*, *Piper cubeba* L.f., *Cinnamomum cassia* (L.) D. Don, *Syringa oblata* Lindl., *Magnolia officinalis Rehd.et Wils., Platycodon grandiflorus* (Jacq.) A.DC, *Citrus reticulata Blanco*, *Sparganium stoloni erum,* Buch. -Ham., *Glycyrrhiza uralensis* Fisch., *Cyperus rotundus* L	Deficiency of spleen and stomach, stabbing pain of chest and abdomen, dilatation of both sides of the chest, dizziness, fatigued cumbersome limbs, fever, diarrhea	Dou (2015)
Liang Fang Ji Ye	Liang Fu Pill	*A. officinarum*, *Cyperus rotundus* L	liver depression and Qi stagnation, stomach cold coagulation, epigastric pain	Xie (1990)
Bei Ji Qian Jin Yao Fang	Gao Liang Jiang Decoction	*A. officinarum*, *Magnolia officinalis Rehd.et Wils.*, *Angelica sinensis* (Oliv.) Diels, Guixin	A sudden cramp in the chest and abdomen, the unbearable boredom of both costal branches	Sun (1955)
Yi Xue Fa Ming	Tian Tai Wu Yao San	*A. officinarum*, *Lindera aggregata* (Sims) Kosterm., *Aucklandia costus* Falc., *Foeniculum vuLgare Mill.*, *Citrus reticulata Blanco*, *Areca catechu* L., *MeLia toosendanSieb.et Zucc.*, *Croton tiglium* L	Cold coagulation and Qi stagnation of liver meridian	Li (1959)
Shi Zhai Bai Yi Xuan Fang	Qun Xun San	*A. officinarum*, *Buthus martensii* Karsch	Wind-toothache, swelling and pain of cheek	Wang (2003)
Sheng Ji Zong Lu	Wa Na Qi San	*A. officinarum*, *Callorhimus ursinus* Linnaeus, *Tetradium ruticarpum* (A. Juss.) T. G. Hartley, *Nardostachys jatamansi* (D. Don) DC., *Citrus reticulata Blanco*	Deficiency of the kidney, Qi deficiency of heart and spleen, intolerable cold pain of small intestine	Zhao (2018)

## 3 Chemical composition

Up to now, 337 chemical compounds have been extracted from *A. officinarum*, mainly including flavonoids, diarylheptanoids, phenylpropanes, glycosides, volatile oil, and other compounds.

### 3.1 Flavonoids

**FIGURE 1:**
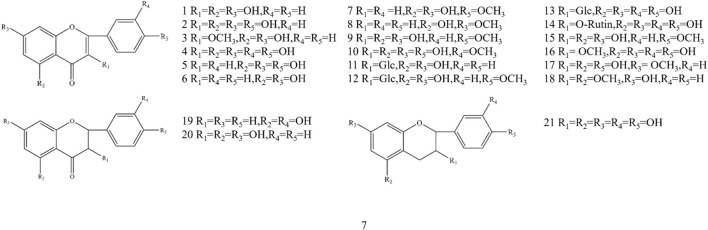
The structure of flavonoids in *A. officinarum*.

Flavonoid is one of the main components in *A. officinarum*. A large number of flavonoids were isolated from *A. officinarum*, which are also the main active components in it. Until now, 21 flavonoids have been isolated, including 18 flavones, 2 flavanones, and 1 flavanol, as shown in Figure 1 and Table 3.

**TABLE 3:** The flavonoids of *A. officinarum*.

NO.	Compound	Reference	NO.	Compound	Reference
1	Galangin	An et al., 2006a; An (2008), Guo et al. (2010)	12	Kaempferide-4′-methylether-3-glucopyranoside	An (2008)
2	Kaempferol	Bu et al. (2000)	13	Isoquercitrin	Wei et al. (2018)
3	Galangin-3-O-methylether	An et al. (2006a)	14	Rutin	Tan et al. (2015)
4	Quercetin	An (2008)	15	Kaempferide-4′-O-methylether	Bu et al. (2000)
5	Apigenin	Zhao et al. (2007)	16	Quercetin-3-O-methylether	Guo et al. (2010)
6	Chrysin	Tan et al. (2015)	17	Rhamnocitrin	Bleier and Chirikdjian (1972), Shen et al., 1998; An (2008)
7	Acacetin	Tan et al. (2015)	18	7-hydroxy-3,5-dimethoxyflavone	Guo et al. (2010)
8	Tectochrysin	Tan et al. (2015)	19	Pinocembrin	An et al. (2006a)
9	Kaempferide	Tan et al. (2015)	20	Dihydrogalanginol	Tushar et al. (2010)
10	Isorhamnetin	Bleier and Chirikdjian (1972)	21	Catechin	Zhao (2018)
11	Galangin 3-O-glucoside	An (2008)			

### 3.2 Diarylheptanoids

**FIGURE 2:**
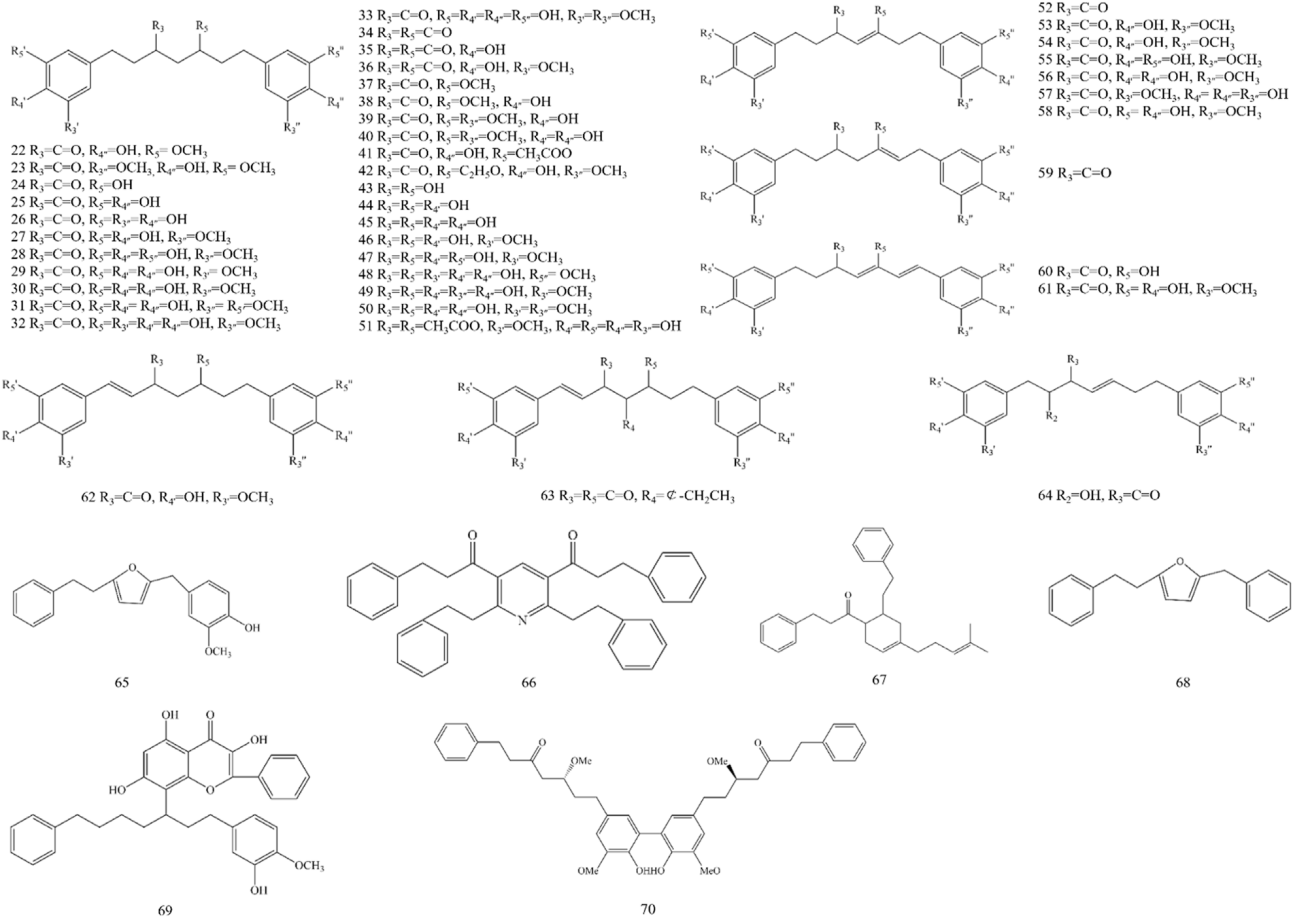
Structure of diarylheptanoids in *A. officinarum*.

Diarylheptanoid is a group of compounds that contain a 1,7-disubstituted aromatic ring and a heptane skeleton and is an important chemical component of *A. officinarum*. At present, 49 diarylheptanoid compounds have been isolated from *A. officinarum*, including 42 chain diarylheptanoids, six cyclic diarylheptanoids, and one polymer of diarylheptanoid and flavonoid, as shown in Figure 2 and Table 4.

**TABLE 4:** The structure of diarylheptanoids in *A. officinarum*.

NO.	Compound	Reference	NO.	Compound	Reference
22	1-phenyl-7-(4″-hydroxyphenyl)-3-heptanone	Hideji et al. (1985)	47	1-(4′,5′-dihydroxy-3′-methoxyphenyl)-7-phenyl 3,5-heptanediol	An (2008)
23	1-phenyl-7-(4″-hydroxy-3″-methoxyphenyl)-3-heptanone	Kiuchi et al. (1992)	48	(3R,5R)-1-(3′,4′-dihydroxyphenyl)-7-(4″-hydroxyphenyl)-3,5-heptanediol	An (2008)
24	5-hydroxy-1,7-bisphenyl-3-heptanone	Sun et al. (2008)	49	(3R,5R)-1-(4′-hydroxy-3′-methoxyphenyl)-7-(3″,4″-dihydroxyphenyl)-3,5-heptanediol	Tian et al. (2009)
25	5-hydroxy-1-phenyl-7 - (4″-hydroxyphenyl)-3-heptanone	An et al. (2006b)	50	1,7-bis-(4′-hydroxy-3′-methoxyphenyl)-3,5-heptanediol	Tian et al. (2009)
26	5-hydroxy-1-phenyl-7 - (3″, 4″-dihydroxyphenyl)-3-heptanone	Sawamura et al. (2010)	51	3,5-diacetoxy-1-(4′,5′-dihydroxy-3′-methoxyphenyl)-7-(3″,4″- dihydroxyphenyl)-heptane	An (2008)
27	5-hydroxy-1-phenyl-7-(4″- hydroxy-3″-methoxyphenyl)-3-heptanone	Matsuda et al. (2009)	52	1,7-diphenyl-4-hepten-3-one	An (2008)
28	5-hydroxy-1-phenyl-7-(4″,5″-dihydroxy-3″-methoxyphenyl)-3-heptanone	Tian et al. (2009)	53	1-phenyl-7-(4″-hydroxyphenyl)-4-hepten-3-one	Sun et al. (2008)
29	5-hydroxy-1-(4′-hydroxy-3′-methoxyphenyl)-7-(4″-hydroxyphenyl)-3-heptanone	Sun et al. (2008)	54	1-phenyl-7-(4″-hydroxy-3″-methoxyphenyl)-4-hepten-3-one	Sun et al. (2008)
30	5-hydroxy-1-(4′-hydroxyphenyl)-7-(4″-hydroxy-3″-methoxyphenyl)-3-heptanone	Shin et al. (2002)	55	1-phenyl-7-(4″,5″-dihydroxy-3″-methoxyphenyl)-4-hepten-3-one	Sawamura et al. (2010)
31	5-hydroxy-1,7-bis - (4′- hydroxy-3′-methoxyphenyl)-3-heptanone	Hideji et al. (1985)	56	1-(4′-hydroxyphenyl)-7-(4″-hydroxy-3″-methoxyphenyl)-4-hepten-3-one	Sawamura et al. (2010)
32	5-hydroxy-1-(3′,4′-dihydroxyphenyl)-7-(4″-hydroxy-3″- methoxyphenyl)-3-heptanone	Tian et al. (2009)	57	1-(4′-hydroxy-3′-methoxyphenyl)-7-(3″,4″-dihydroxyphenyl)-4-hepten-3-one	Tian et al. (2009)
33	5-hydroxy-1-(4′-hydroxy-3′-methoxyphenyl)-7-(4″,5″-dihydroxy-3″-methoxyphenyl)-3-heptanone	An (2008)	58	5-hydroxy-1-phenyl-7-(4″-hydroxy-3″-methoxyphenyl)-4-hepten-3-one	An (2008)
34	1,7-diphenyl-3,5-heptanedione	An (2008)	59	1,7-diphenyl-5-hepten-3-one	Zhang et al. (2010)
35	1- (4′-hydroxyphenyl)-7-phenyl-3,5-heptanedione	An (2008)	60	5-hydroxy-1,7-bisphenyl-4,6-heptadien-3-one	An et al. (2006b)
36	1-(4′-hydroxy-3′-methoxyphenyl)-7-phenyl-3,5-heptanedione	An (2008)	61	5-hydroxy-1-(4′-hydroxy-3′-methoxyphenyl)-7-phenyl-4,6-heptadiene-3-one	An et al. (2006b)
37	5-methoxy-1,7-bisphenyl-3-heptanone	Tian et al. (2009)	62	1-(4′-hydroxy-3′-methoxyphenyl)-7-phenyl-1-hepten-3-one	Kiuchi et al. (1992)
38	5-methoxy-1-phenyl-7-(4″-hydroxyphenyl)-3-heptanone	Tian et al. (2009)	63	4-phenethyl-1,7-bisphenyl-1-heptene-3,5-dione	Zhang et al. (2010)
39	5-methoxy-1-phenyl-7-(4″-hydroxy-3″-methoxyphenyl)-3-heptanone	Tian et al. (2009)	64	2-hydroxy-1,7-bisphenyl-4-hepten-3-one	Sun et al. (2008)
40	5-methoxy-1-(4′-hydroxyphenyl)-7-(4″-hydroxy-3″-methoxyphenyl)-3-heptanone	Tian et al. (2009)	65	officinarumane A	An (2008)
41	5-acetoxy-1-phenyl-7-(4″-hydroxyphenyl)-3-heptanone	An (2008)	66	officinarumane B	An (2008)
42	5-ethoxy-1-phenyl-7- (4″-hydroxy-3″-methoxyphenyl)-3 heptanone	Liu and Liu (2016)	67	officinarumane C	An (2008)
43	1,7-diphenyl-3,5-heptanediol	Matsuda et al. (2009)	68	2-benzyl-5-phenylethyl furan	Shen et al. (1998)
44	(3R,5R)-1-(4′-hydroxyphenyl)-7-phenyl-3,5-heptadiol	Uehara et al. (1987)	69	officinin	Wei et al. (2016)
45	(3R,5R)-1,7-bis-(4′-hydroxyphenyl)-3,5-heptanediol	Tian et al. (2009)	70	(5R,5′R)-7,7'-(6,6′-dihydroxy-5,5′-dimethoxy [1,1′-biphenyl]-3,3′-diyl)bis [5-methoxy-1-phenylheptan-3-one]	Sun et al. (2008)
46	1-(4′-hydroxy-3′-methoxyphenyl)-7-phenyl-3,5-heptanediol	Tian et al. (2009)			

### 3.3 Volatile oil

*A. officinarum* is a type of pungent and warm botanical drugs with a high content of volatile oil. Its spicy scent is one of the indicators used to judge the quality of this herbal medicine. At present, 241 volatile oils have been separated from *A. officinarum*, mainly including terpenoids (monoterpenes, sesquiterpenoids), aldehydes, ketones, ethers, alcohols, phenols, and other compounds, as shown in Table 5.

**TABLE 5:** Volatile oil from *A. officinarum*.

NO.	Compound name	Reference	NO.	Compound name	Reference
71	1,8- Eucalyptol	Gao et al. (2012), Dong and Cai (2015), Zou et al. (2018)	192	isopentyl isovalerate	Zhai et al. (2014a), Zou et al. (2018)
72	camphene hydrate	Dong and Cai (2015), Zou et al. (2018)	193	2-methylbutyl valerate	Zou et al. (2018)
73	(+)-borneol	Gao et al. (2012), Zhai et al. (2014a)	194	2-methylbutyric acid-3-methylbutyl ester	Zou et al. (2018)
74	Isoborneol	Zou et al. (2018)	195	linalyl acetate	Gao et al. (2012)
75	alpha-terpineol	Yuan et al. (2016), Zou et al. (2018)	196	isobutyl 2-methylbutyrate	Zhai et al. (2014a), Zou et al. (2018)
76	Borneol	Zou et al. (2018)	197	cis-3-hexenyl acetate	Zhai et al. (2014a)
77	β-pinene	Dong and Cai (2015), Zou et al. (2018)	198	bornyl acetate	Gao et al. (2012), Zou et al. (2018)
78	camphene	Zhai et al. (2014a), Dong and Cai (2015), Zou et al. (2018)	199	benzaldehyde	Dong and Cai (2015)
79	terpinolene	Dong and Cai (2015), Yuan et al. (2016), Zou et al. (2018)	200	phenylpropanal	Dong and Cai (2015), Tang et al. (2021)
80	alpha-fenchene	Dong and Cai (2015)	201	5-hydroxymethylfurfural	Dong and Cai (2015)
81	gamma-terpinene	Gao et al. (2012), Dong and Cai (2015), Yuan et al. (2016)	202	uronic acid	Zou et al. (2018), Tang et al. (2021)
82	Pinene	Zou et al. (2018)	203	sweet neral	Yuan et al. (2016)
83	(R)-(+)-limonene	Zou et al. (2018)	204	1,1-diethoxyethane	Tang et al. (2021)
84	(+)-3-carene	Zou et al. (2018)	205	p-methylphenyl isopropanol	Dong and Cai (2015), Zou et al. (2018)
85	alpha-terpinene	Gao et al. (2012), Zhai et al. (2014a), Yuan et al. (2016)	206	4-phenyl-2-butanol	Zou et al. (2018)
86	3-carene	Zou et al. (2018)	207	1-methyl-4-(1-methylvinyl)cyclohexanolc	Zou et al. (2018)
87	tricyclo [2.2.1.0 (2,6)]heptane,1,7,7-trimethyl-	Zhai et al. (2014a), Zou et al. (2018)	208	octatriacontyl trifluoroacetate	Zou et al. (2018)
88	phellandrene	Zou et al. (2018)	209	(−)-4-terpineol	Dong and Cai (2015), Zou et al. (2018)
89	(−)-α-pinene	Zhai et al. (2014a), Yuan et al. (2016), Zou et al. (2018)	210	cuminol	Gao et al. (2012), Zou et al. (2018)
90	3,7-dimethyl-1,3,6-octatriene	Dong and Cai (2015), Zou et al. (2018)	211	2,6,6-trimethyl-bicyclo [3.1.1]heptane-2,3-diol	Gao et al. (2012)
91	limonene	Gao et al. (2012), Yuan et al. (2016)	212	2,3-butanediol	Tang et al. (2021)
92	beta-phellandrene	Gao et al. (2012)	213	alpha-juniperol	Gao et al. (2012), Yuan et al. (2016)
93	(−)-camhene	Zou et al. (2018)	214	linalool	Yuan et al. (2016), Zou et al. (2018)
94	α-thujene	Yuan et al. (2016)	215	trans-rosinol	Yuan et al. (2016)
95	β-pinene	Yuan et al. (2016)	216	(Z)-linalool oxide	Yuan et al. (2016)
96	β-myrcene	Yuan et al. (2016)	217	L-linalool	Yuan et al. (2016)
97	2, 6-dimethyl-1, 3, 7-octadiene	Zhai et al. (2014a)	218	2,3-butanediol	Tang et al. (2021)
98	(+)-M-mentha-1.8-diene	Zhai et al. (2014a)	219	camphor	Dong and Cai (2015), Yuan et al. (2016), Zou et al. (2018)
99	(+)-4-carene	Zou et al. (2018)	220	benzyl acetone	Dong and Cai (2015), Zou et al. (2018), Tang et al. (2021)
100	O-cymene	Zou et al. (2018)	221	methylheptenone	Zhai et al. (2014a), Zou et al. (2018)
101	valencia orangeene	Gao et al. (2012), Dong and Cai (2015)	222	5-hydroxymethyl-2(5H)-furanone	Tang et al. (2021)
102	1-caryophyllene	Gao et al. (2012), Dong and Cai (2015), Yuan et al. (2016)	223	(1S)-(−)-camphor bicyclo [2.2.1]heptan-2-one,1,7,7-trimethyl-, (1S)	Zou et al. (2018)
103	γ-elemene	Dong and Cai (2015)	224	4-methyl-5-nonanone	Zou et al. (2018)
104	(+)-fumene	Gao et al. (2012), Dong and Cai (2015)	225	3-butylene-1(3H)-isobenzofuranone	Dong and Cai (2015)
105	α-farnesene	Dong and Cai (2015), Yuan et al. (2016)	226	6-methyl-5-hepten-2-one	Yuan et al. (2016)
106	(−)-β-huperene	Tang et al. (2021)	227	2-methoxy-4-vinylphenol	Tang et al. (2021)
107	longifolene	Gao et al. (2012), Dong and Cai (2015)	228	paeonol	Dong and Cai (2015)
108	phenylethanol	Tang et al. (2021)	229	2,6-di-tert-butyl-p-cresol	Zou et al. (2018)
109	γ-muurolene	Yuan et al. (2016), Zou et al. (2018)	230	2,2′-methylenebis (6-tert-butyl-p-cresol)	Zou et al. (2018)
110	naphthalene,1,2,3,4,4a,5,6,8a-octahydro-4a,8-dimethyl-2-(1-methylethenyl)-, [2R-(2α,4aα,8aβ)]	Zou et al. (2018)	231	naphthalene,1,2,3,4,4a,5,6,8a-octahydro-7-methyl-4-methylene-1-(1-methylethyl)-, (1α,4aβ,8aα)	Zou et al. (2018)
111	γ-selinene	Gao et al. (2012), Zou et al. (2018)	232	4-ethyl-2-methoxyphenol	Gao et al. (2012)
112	(E)-alpha-bergamotene	Zou et al. (2018)	233	hirsutene	Yuan et al. (2016)
113	1,6-cyclodecadiene,1-methyl-5-methylene-8-(1-methylethyl)-, [S-(E,E)]	Zou et al. (2018)	234	(1,7,7-trimethylnorbornane-2-YL) acetate bicyclo [2.2.1]heptan-2-ol,1,7,7-trimethyl-, 2-acetate	Zou et al. (2018)
114	(−)-alpha-piperolene	Yuan et al. (2016), Zou et al. (2018)	235	AR-curcumene	Zhai et al. (2014a)
115	α-elemene	Zou et al. (2018)	236	3,5-dimethoxytoluene	Dong and Cai (2015)
116	2,6-dimethyl-6-(4-methyl-3-pentenyl) bicyclo [3.1.1]hept-2-ene	Zou et al. (2018)	237	safrole	Dong and Cai (2015)
117	(−)-alpha-gurenene	Gao et al. (2012), Dong and Cai (2015)	238	palmitic acid	Dong and Cai (2015)
118	α-amorphene	Zou et al. (2018)	239	acetamic acid	Tang et al. (2021)
119	α-caryophyllene	Dong and Cai (2015), Yuan et al. (2016)	240	3,6-dimethyl-2,3,3a,4,5,7a-hexahydrobenzofuran	Zou et al. (2018)
120	α-Ilanolene	Gao et al. (2012)	241	1,2,3,4-tetrahydronaphthalene	Zou et al. (2018)
121	(−)-isosativene	Gao et al. (2012)	242	anethole	Zou et al. (2018)
122	isoflavene	Gao et al. (2012)	243	octadecane	Zou et al. (2018)
123	α-longleaf pinene	Gao et al. (2012)	244	nonadecane	Dong and Cai (2015), Zou et al. (2018)
124	α-guaiene	Gao et al. (2012)	245	eicosan	Zou et al. (2018)
125	γ-gurenene	Gao et al. (2012)	246	hecosane	Zou et al. (2018)
126	beta-juniperene	Gao et al. (2012)	247	docosane	Zou et al. (2018)
127	aristolochene	Gao et al. (2012)	248	pentacosane	Zou et al. (2018)
128	(+)-hornene	Gao et al. (2012)	249	trisane	Zou et al. (2018)
129	epizonarene	Gao et al. (2012)	250	tetracosane	Zou et al. (2018)
130	β-cedrene	Gao et al. (2012)	251	hexadecane	Zou et al. (2018)
131	delta-juniperene	Gao et al. (2012), Yuan et al. (2016)	252	1-docosene	Zou et al. (2018)
132	calamene	Gao et al. (2012)	253	cholesta-3,5-diene	Zou et al. (2018)
133	alpha-elemene	Gao et al. (2012)	254	2,3-dihydrobenzofuran	Tang et al. (2021)
134	(−)-isoprene	Gao et al. (2012)	255	2,4-cyclohexadien-1-one,3,5-bis(1,1-dimethylethyl)-4-hydroxy-	Zou et al. (2018)
135	neosyringatricyclone	Gao et al. (2012)	256	2,4-dimethylbenzo[h]quinoline	Zou et al. (2018)
136	ylangene	Yuan et al. (2016)	257	toluene	Zou et al. (2018)
137	α-copaene	Zhai et al. (2014a), Yuan et al. (2016)	258	2,4-dimethylstyrene	Dong and Cai (2015)
138	β-elemene	Yuan et al. (2016)	259	(1R,2S,3S)-1,2-dimethyl-3-isopropenylcyclopentanol	Zou et al. (2018)
139	santalene	Yuan et al. (2016)	260	t-cadinol	Zou et al. (2018)
140	trans-bergamotene	Yuan et al. (2016)	261	β-santalol	Gao et al. (2012)
141	fragranene	Yuan et al. (2016)	262	pentanoic acid,2-ethylhexyl ester	Zou et al. (2018)
142	geranene D	Yuan et al. (2016)	263	decane, 3,3,6-trimethyl-	Zou et al. (2018)
143	cyclic isofolene	Yuan et al. (2016)	264	2-dodecen-1-yl succinic anhydride	Zou et al. (2018)
144	beta-selinene	Yuan et al. (2016)	265	2-octylcyclopropaneoctanal	Zou et al. (2018)
145	β-bisabolene	Yuan et al. (2016)	266	3-methyloctadecane	Zou et al. (2018)
146	β-panasinsene	Yuan et al. (2016)	267	1H-pyrrole, 1-butyl-	Zou et al. (2018)
147	γ-cadinene	Yuan et al. (2016)	268	bergamotenol	Zou et al. (2018)
148	selina-3,7 (11)-diene	Yuan et al. (2016)	269	methyl eugenol	Zou et al. (2018)
149	germacrene B	Yuan et al. (2016)	270	2-hydroxy-1,8-cineole	Yuan et al. (2016)
150	allomanerene	Zhai et al. (2014a)	271	(E)-linalool oxide (furanoid)	Zhai et al. (2014a)
151	α-amorphene	Zhai et al. (2014a)	272	(cis)-2-methyl-2-vinyl-5-isopropyl-tetrahydrofuran	Zhai et al. (2014a)
152	caryophyllene oxide	Dong and Cai (2015), Yuan et al. (2016)	273	juniper camphor	Zou et al. (2018)
153	2-methyl-1-propanol butyrate	Dong and Cai (2015)	274	α-bergamotol	Yuan et al. (2016)
154	bornyl L-acetate	Zhai et al. (2014a), Dong and Cai (2015)	275	5-hydroxy-1,7-diphenyl-3-heptanone	Yuan et al. (2016)
155	acetate-(4-phenyl)-2-butyl ester	Dong and Cai (2015)	276	(E,E)-2,6-dimethyl-2,6-octadienedial	Zhai et al. (2014a)
156	methyl cinnamate	Dong and Cai (2015)	277	3-methylene-6-hepten-2-one	Zhai et al. (2014a)
157	methyl isovalerate	Dong and Cai (2015)	278	1-cmethylene-6-hepten-2-one	Zhai et al. (2014a)
158	isobutyl isobutyrate	Zhai et al. (2014a), Zou et al. (2018)	279	1-nonyne	Zhai et al. (2014a)
159	fenugreek acetate	Dong and Cai (2015), Zou et al. (2018), Tang et al. (2021)	280	4-(2-oxopropyl) cycloheptan-1-one	Zhai et al. (2014a)
160	2-phenylethyl isobutyrate	Yuan et al. (2016), Zou et al. (2018)	281	longanine	Zhai et al. (2014a)
161	methyl myristate	Zou et al. (2018)	282	benzoic acid,2,4-bis [(trimethylsilyl) oxy]-, teimethysilyl ester	Zou et al. (2018)
162	phenethyl butyrate	Zou et al. (2018)	283	octadecylcyclononane siloxane	Zou et al. (2018)
163	ethylene glycol dimethacrylate	Zou et al. (2018)	284	borneol chloride	Gao et al. (2012)
164	isobutyl isovalerate	Yuan et al. (2016), Zou et al. (2018)	285	1-chlorodocosane behenyl chloride	Zou et al. (2018)
165	isoamyl isobutyrate	Zhai et al. (2014a), Yuan et al. (2016), Zou et al. (2018)	286	butyl isothiocyanate	Zou et al. (2018)
166	amyl valerate	Zou et al. (2018)	287	2-bromo-4,5-dimethoxycinnamic acid	Zou et al. (2018)
167	isobutyl benzoate	Yuan et al. (2016), Zou et al. (2018)	288	2-benzylimidazoline	Zou et al. (2018)
168	2-phenylethyl isovalerate	Zhai et al. (2014a), Yuan et al. (2016), Zou et al. (2018)	289	cyclopentacarbazide	Zou et al. (2018)
169	2-methylbutyric acid-2-phenethyl ester	Zou et al. (2018)	290	4-amino-5-cyano-7-(beta-d-ribofuranose) pyrrolo [2,3-day] pyrimidine toyocamycin	Zou et al. (2018)
170	N-phenylacetamide	Gao et al. (2012)	291	10S,11S-cedar-3 (12)-diene	Dong and Cai (2015)
171	isocineole	Gao et al. (2012)	292	androst-5,15-dien-3ol acetate	Zou et al. (2018)
172	1-methyl-4-(1-methylethyl)-1,3-cyclohexadiene	Gao et al. (2012)	293	borneol, trifluoroacetate	Zou et al. (2018)
173	aminobenzyl alcohol	Gao et al. (2012)	294	benzamide,2,3,4-trifluoro-N-methyl-N-phenyl-	Zou et al. (2018)
174	Seselene	Gao et al. (2012)	295	bicyclo [2.2.1]heptane,2-chloro-1,7,7-trimethyl-, (1R-endo)-	Zou et al. (2018)
175	trans-1,3-diphenylcyclobutane	Gao et al. (2012)	296	1,3,5,7,9-pentaethylbicyclo [5.3.1] pentasiloxane	Zou et al. (2018)
176	β-chlorophylene	Gao et al. (2012)	297	silane, (2-ethynylphenyl) trimethyl-	Zou et al. (2018)
177	1,4-bis [methyl (tetramethylene)silyloxy]butane	Gao et al. (2012)	298	1-cyano-N-fluoroformimidoyl fluoride (anti)	Zou et al. (2018)
178	citronella	Gao et al. (2012)	299	3,6-dimethyl-2,3,3a,4,5,7a-hexahydrobenzofuran	Zou et al. (2018)
179	3,7 (11)-epipindiene	Gao et al. (2012)	300	ethyl 2-(5-methyl-5-vinyltetrahydrofuran-2-yl) propan-2-yl carbonate	Zou et al. (2018)
180	thornene	Gao et al. (2012)	301	3-quinolinecarboxylic acid,6,8-difluoro-4-hydroxy-, ethyl ester	Zou et al. (2018)
181	spiroterpene alcohol	Gao et al. (2012)	302	trimethylsilyl 3-methyl 4-[(trimethylsilyl) oxy] benzoate	Zou et al. (2018)
182	(1α,4aα,8aα)-1,2,3,4,4a,5,6,8a-octahydro-7-methyl-4-methylene-1-(1-methylethyl)-naphthalene	Gao et al. (2012)	303	oxirane,2-methyl-3-phenyl-	Zou et al. (2018)
183	3,8-dimethyl-5-(1-methylethyl)-1,2-naphthalenedione	Gao et al. (2012)	304	cyclobutanecarboxylic acid,2-phenylethyl ester	Zou et al. (2018)
184	(4aR-trans)-1,2,3,4,4a,5,6,8a-octahydro-4a,8-dimethyl-2-(1-methylethylene)-naphthalene	Gao et al. (2012)	305	4-nitrobenzoylmethyl-β-phenylpropionate	Zou et al. (2018)
185	γ-longleaf pinene	Gao et al. (2012)	306	di-epi-α-ccedrene-(Ⅰ)	Zou et al. (2018)
186	alpha-santalol	Gao et al. (2012)	307	ar-tumerone	Zou et al. (2018)
187	1,4-dimethyl-7-(1-methylethyl)-chamomile	Gao et al. (2012)	308	tetrapentacontane,1,54-dibromo-	Zou et al. (2018)
188	decane,5,6-bis(2,2-dimethylpropylidene)-(Z,Z)	Zou et al. (2018)	309	e−8-methyl-9-tetradecen-1-ol acetate	Zou et al. (2018)
189	1,1,6-trimethyl-3-methylene-2 (3,6,9,13-tetramethyl-6-ethenye-10,14-dimethylene-pentadec-4-enyl)cyclohexane	Zou et al. (2018)	310	hexadecanediniteile	Zou et al. (2018)
190	sulfurous acid, butyl heptadecyl ester	Zou et al. (2018)	311	octacosyl trifluoroacetate	Zou et al. (2018)
191	cyclohexane,1,2-dimethyl-3-pentyl-4-propyl	Zou et al. (2018)			

### 3.4 Other compounds

**FIGURE 3:**
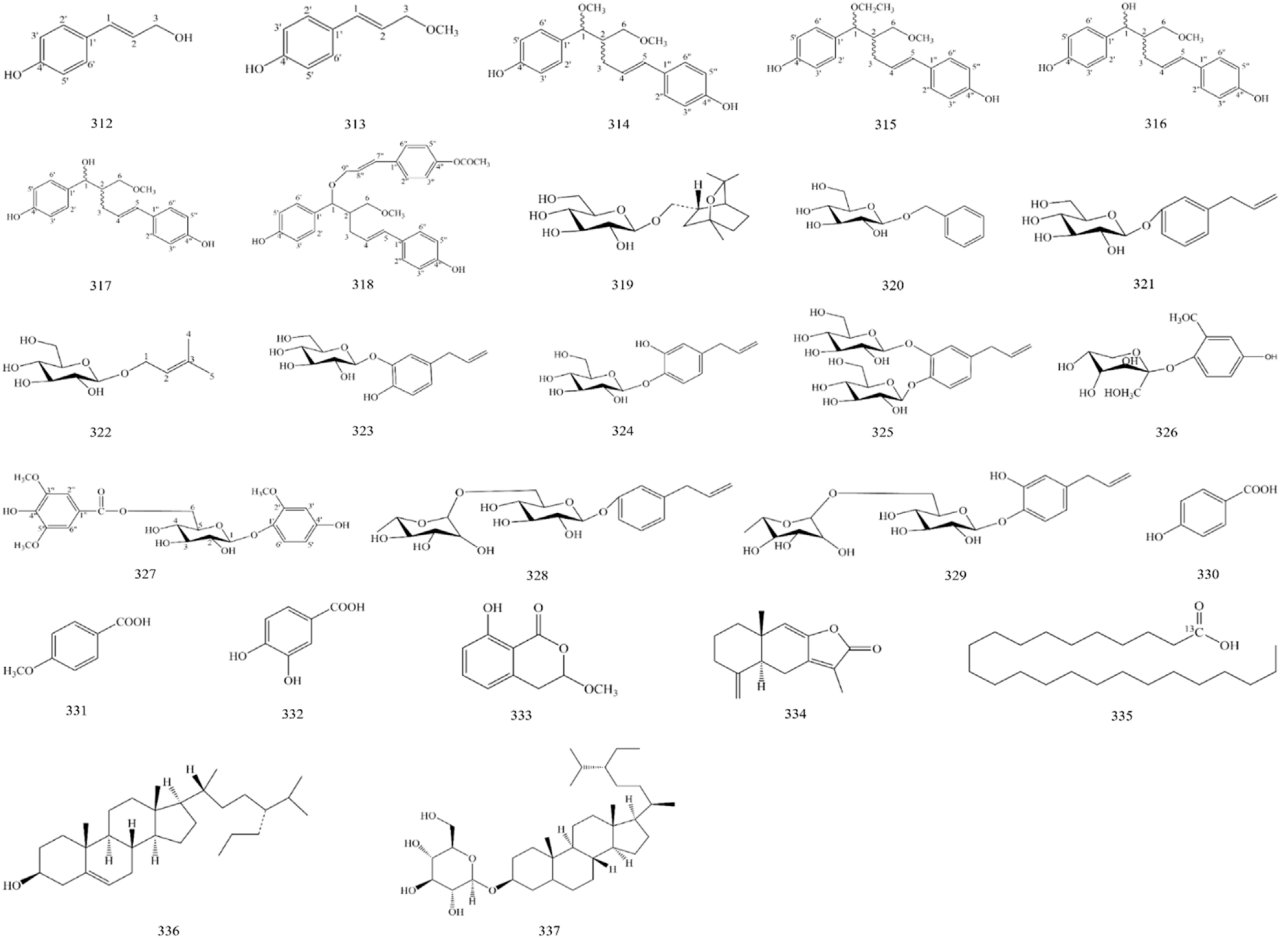
Structure of other compounds in *A. officinarum*.

In addition, *A. officinarum* contains 7 phenylpropanoids, 11 glycosides, 5 organic acids, 2 sterols and their glycosides, and 1 lactone, as shown in Figure 3 and Table 6.

**TABLE 6:** Other compounds in *A. officinarum*.

NO.	Compound	Category	Reference	NO.	Compound	Category	Reference
312	p-hydroxyphenylpropenol	phenylpropanoid	Ly et al. (2003)	325	1,2-bis-O-β-D-glucopyranosyl-4-allylbenzene	glycoside	Ly et al. (2002)
313	p-hydroxyphenylpropene methyl ester	phenylpropanoid	Ly et al. (2003)	326	N-butyl-β-D-fructopyranoside	glycoside	Ly et al. (2002)
314	(4E)-1,5-bis-(4-hydroxyphenyl)-1-methoxy-2-(methoxy)-phenyl-4-pentene (2a,2b)	phenylpropanoid	Ly et al. (2003)	327	4′-hydroxy-2′-methoxyphenol	glycoside	Ly et al. (2002)
315	(4E)-1,5-bis-(4-hydroxy)-phenyl-2-(methoxymethyl)4-penten-1-ol (2a,2b)	phenylpropanoid	Ly et al. (2003)	328	1-O-(6-Oα-L-rhamnopyranosyl-β-D-glucopyranosyl)-4-allylbenzene	glycoside	Ly et al. (2002)
316	(4E)-1,5bis-(4-hydroxyphenyl)-1-ethoxy-2-(methoxymethyl)-4-pentene (2a,2b)	phenylpropanoid	Ly et al. (2003)	329	1-O-(6-O-α-L-rhamnopyranosyl-β-D-glucopyranosyl)-2-hydroxy-4-allylbenzene	glycoside	Ly et al. (2002)
317	(4E)-1,5-bis-(4-hydroxy)-phenyl-2-(hydroxy)-phenyl-4-penten-1-ol (2a,2b)	phenylpropanoid	Ly et al. (2003)	330	p-hydroxybenzoic acid	organic acid	An (2008)
318	(4E)-1,5-bis-(4-hydroxy)-phenyl-1-[(2E)-3-(4-acetoxyphenyl)-2-propenyloxy]-2-diethyl ether-4-pentene	phenylpropanoid	Ly et al. (2003)	331	p-methoxybenzoic acid	organic acid	An (2008)
319	(1R,3S,4S)-trans-3-hydroxy-1,8-cineole-β-D-glucopyranoside	glycoside	An et al. (2006c)	332	3,4 dihydroxybenzoic acid	organic acid	An (2008)
320	benzyl-β-D-glucopyranoside	glycoside	An et al. (2006c)	333	8-hydroxy-3-methoxyisochroman-1-one	organic acid	An (2008)
321	1-O-β-D-glucopyranosyl-4-allylbenzene	glycoside	Ly et al. (2002)	334	behenic acid	organic acid	An (2008)
322	3-methyl-2-butene-β-D-glucopyranoside	glycoside	Ly et al. (2002)	335	atractylide Ⅰ	lactone	An (2008)
323	1-hydroxy-2-O-β-D-glucopyranosyl-4-allylbenzene	glycoside	Ly et al. (2002)	336	beta-sitosterol	sterol	An (2008)
324	1-O-β-D-glucopyranosyl-2-hydroxy-4-allylbenzene	glycoside	Ly et al. (2002)	337	carotene	terpenoid	An (2008)

## 4 Pharmacokinetic study of the active compounds of *A. officinarum*

As one of the main active compounds of *A. officinarum*, galangin (3,5,7-trihydroxyflavone) has a variety of biological activities. Once galangin is consumed, it is metabolized in the intestine and liver, where it undergoes glucuronidation, methylation, and sulfation reactions. The pharmacokinetics of galangin-3-O-β-D-glucuronic acid (GG-1) and galangin-7-O-β-D-glucuronic acid (GG-2), two metabolites of *A. officinarum*, were studied *in vivo*. It was found (Liu et al., 2021) that, after oral administration of *A. officinarum* extract (0.3 g/kg) in rats, the peak concentrations (C_max_) of GG-1 and GG-2 were 6069.6 ± 1140.6 and 10596.0 ± 2395.7 ng/mL, respectively, reaching their peak concentrations at 0.2 ± 0.1 h. Area under curve (0-t) (AUC_0-t_), mean residence time (0-t) (MRT_0-t_), and t_1/2_ of GG-1 were 2390.9 ± 678.0 h μg/L, 1.4 ± 0.8 h, and 2.2 ± 0.7 h, respectively, while the corresponding values of GG-2 were 4554.9 ± 884.9 h·μg/L, 1.6 ± 0.7 h, and 3.3 ± 0.2 h, respectively. Obviously, the most significant difference between GG-1 and GG-2 is the AUC0-t and Cmax, where the parameter values of GG-2 are almost twice those of GG-1.

In addition, a previous study (Xin Zhang et al., 2021) found that microemulsion can promote the absorption of galangin and improve its bioavailability. The blood concentration of galangin in Liangfu Pill could not be detected after the rabbits were given Liangfu Pill by gavage once. For Liangfu micromilk, the absorption half-life (t_1/2ka_) of galangin was 0.29 h, the peak time (t_peak_) was 0.75 h, the elimination half-life (t_1/2ke_) was 1.47 h, C_max_ was 38.46 μg/L, and the AUC was 129.42 (μg·h)/L. In another study (Xianhua Du et al., 2008), it was found that a self-microemulsion of galangin was absorbed throughout the entire intestinal tract of rats. The absorption rate constants (K_a_) in the duodenum, jejunum, ileum, and colon were 2.37, 1.70, 2.29, and 3.98 times higher than those of the galangin suspension, respectively. Additionally, the apparent absorption coefficients (Papp) were 3.58, 2.56, 3.57, and 5.16 times higher than those of the galangin suspension, respectively. The relative bioavailability of the self-microemulsion of galangin was 220%, compared to the galangin suspension.

## 5 Pharmacological effects of *A. officinarum*

*A. officinarum* is an important traditional Chinese medicine, and its main chemical components are flavonoids, volatile oils, and diarylheptanoids. Modern pharmacological studies have shown that *A. officinarum* has various pharmacological effects, including anti-ulcer, inhibition of gastrointestinal motility, anti-inflammatory and analgesic, antioxidant, anti-tumor, antibacterial, and hypoglycemic properties, as shown in Table 7.

**TABLE 7:** Study on pharmacological effects of *A. officinarum*.

Pharmacological effects	Extracts/compounds	Model	Dosage/concentration	Effects/mechanisms	Reference
Antiulcer	Supercritical extract of *A. officinarum*	SD rat, model of restraint water immersion stress ulcer	High and low dose group 100, 50, 25 mg/(kg·d), administration for 4 days, once a day	Reducing the ulcer index of rats with restraint water immersion stress ulcer and reducing the gastric juice secretion, serum GAS level and pepsin activity of the model rats, the gastric mucosal SS level increased significantly, approaching the normal level	Peng et al. (2008)
	Supercritical extract of *A. officinarum*	SD rat, model of restraint water immersion stress ulcer	High and low dose group 100, 50, 25 mg/(kg·d), administration for 5 days, once a day	Reduce the ulcer index of the model rats and significantly increase the levels of serum IL-2 and EGF in the model rats, bring them close to normal levels	Wu et al. (2004a)
	Galangin	SD male rats, pylorus ligated gastric ulcers model, indomethacin gastric ulcers model, ICR male mice, ethanol gastric ulcers model	Pylorus ligation gastric ulcers model: 100 mg/kg, once a day for 5 days, indomethacin and ethanol gastric ulcers models: 50, 100, 200 mg/kg, once a day for 6 days	Galangin has an obvious effect on gastric ulcers in mice with alcoholic gastric ulcers induced by pylorus ligation, but it has no effect on the indomethacin gastric ulcers model in rats	Li (2007)
	Different extracts of *A. officinarum*	Kunming mice, SD rats; ethanol-induced gastric mucosal injury model in mice; gastric ulcers model in rats induced by aspirin and indomethacin	0.75, 3.00, 12 g/kg body weight, ethanol model for 7 days, aspirin model for 15 days, indomethacin model for 10 days, once a day	The mechanism of the anti-ulcer effect of *A. officinarum* may be through inhibiting inflammatory factors to decrease GAS and increase COX-2 and PGE2, thereby improving the protective effect of gastric mucosa and reduce the injury of the gastric ulcers	Wei (2019)
	*A. officinarum*	BALB/c mice; animal model of *Helicobacter pylori* associated gastritis	Low, medium and high dose: 0.09 g/kg, 0.18 g/kg, 0.36 g/(kg·day), 21 days	*A. officinarum* may inhibit *H. pylori*--associated gastritis by inhibiting the activation of MAPK and its catalysis of NF-κB phosphorylation	Ma (2019)
	*A. officinarum* oil	ICR mice, reserpine to mouse gastric ulcers model	High, middle and low dose groups: 8, 4 and 2 mL/kg, once a day, for 6 days	*A. officinarum* oil can increase the activity of serum SOD and decrease the level of MDA to play a role in antioxidant stress and achieve the purpose of anti-GU	Wang et al. (2011)
	*A. officinarum* oil	ICR mice, reserpine to mouse gastric ulcers model	High, middle and low dose groups: 8, 4 and 2 mL/kg, once a day, for 6 days	*A. officinarum* oil can relieve spasms of gastrointestinal smooth muscle in mice with gastric ulcers induced by reserpine and reduce tension in gastrointestinal muscles and exert its antispasmodic and analgesic effects	Wang Haiyan et al. (2011)
	diphenylheptane extract of *A. officinarum*	Female BALB/c mice, the model of gastric injury induced by ethanol	High, middle and low dose: 126.8 mg/kg, 63.4 mg/kg, 31.7 mg/kg, given for 7 days	DPHs increased the activity of superoxide dismutase, decreased the levels of inflammatory mediators, malondialdehyde, motilin, and gastrin, decreased the activities of inducible nitric oxide synthase and cyclooxygenase-2, and inhibited the expression of Toll-like receptor 4, myeloid differentiation factor 88 and nuclear factor-κ B on protein and mRNA	Lin et al. (2021)
	Total flavonoids of *A. officinarum*	*In vivo*: BALB/c mice; ethanol-induced gastric ulcers model *in vivo* and *in vitro*; gastric mucosal epithelial cells *in vitro*	High, middle, and low dose: 126.8 mg/kg, 63.4 mg/kg, 31.7 mg/kg	The total flavonoids of *A. officinarum* effectively decreased the ulcer index, decreased the release of inflammatory mediators (IL-1β, IL-6, TNF- α and PGE2), increased the content of nitric oxide, and improved the secretion of GAS and MTL	Lin et al. (2020)
Inhibition of gastrointestinal motility	*A. officinarum* decoction and its different parts	Kunming mouse; New Zealand rabbit	High and low dose: 8 g/kg and 4 g/kg for 7 days	The main antispasmodic and analgesic components of *A. officinarum* are flavonoids and diarylheptanoids, in which the gastrointestinal spasmolysis is stronger than that of flavonoids, and the analgesic effect of diarylheptanoids is stronger	Gui et al. (2021)
	Different active parts of *A. officinarum*	Ten New Zealand rabbits, both male and female	0.05 g/L	The active components of *A. officinarum* extract could inhibit the spontaneous movement of intestinal muscle in a dose-dependent manner and each active component could inhibit intestinal spasm induced by acetylcholine	Cheng et al. (2015)
	*A. officinarum*	New Zealand rabbit; NIH mouse; SD rat	0.2 mL/10 g	The supercritical extract of *A. officinarum* can inhibit the excitation of intestinal smooth muscle induced by neostigmine and antagonize muscarinic receptors	Wu et al. (2004b)
Analgesic and anti-inflammatory	Total flavonoids of *A. officinarum*	NIH mouse; SD rat	Low, medium, and high doses: 16.6, 33.2, and 66.4 g/kg	The total flavonoids of *A. officinarum* had an obvious inhibitory effect on the acute inflammation model and pain in mice induced by acetic acid and heat stimulation	Chen et al. (2009)
	Total flavonoids of *A. officinarum*	SD rats, NIH mice; acetic acid-induced IBS model rats	High, middle, and low dose: 2, 1, 0.5 g/kg, for two consecutive weeks, once a day	The total flavonoids of *A. officinarum* can effectively reduce the visceral sensitivity of IBS rats induced by acetic acid and inhibit the pain induced by heat stimulation, acetic acid, and formaldehyde in mice	Liang et al. (2013)
	Galangin	KM mouse; NRK-52E cell; mouse UAN model	Low, medium and high doses: 100, 200, 400 mg/kg, once a day for 15 days	Galangin can significantly inhibit the activation of NLRP3 inflammasomes and the release of inflammatory factors IL-1β and IL-18 in NRK-52E cells	Lu et al. (2020)
	Galangin	Female BALB/c mice; asthma model	15.5 mg/kg, once a day for 4 days	Galangin can exert its anti-inflammatory effect by inhibiting the activity of NF-κB and down-regulating the expression of MCP-1, Eotaxin, CXCL10, and VCAM-1 mRNA in human airway smooth muscle cells induced by TNF-α	Cha (2015)
	Galangin	Female BALB/c mice; establishment of mouse asthma model sensitized and challenged by ovalbumin	10 mg/kg	Galangin can reduce the expression of TNF-α and decrease airway inflammation in asthmatic mice	Gu and Wu (2017)
	*A. officinarum*	Male SD rats; Kunming mice, half male and half female	Alcohol extract of *A. officinarum* 20 g/kg, 10 g/kg, 5 g/kg, water extract 30 g/kg, 15 g/kg	*A. officinarum* has a certain effect on fever and inflammation, and the 75% ethanol extract has a stronger effect than the water extract	Yan et al. (2013)
	Galangin	Adult male ICR mice; BV2 microglial cell line	50 mg/kg, once a day for 4 days	Galangin inhibits the expression of pro-inflammatory molecules such as inducible nitric oxide synthase (iNOS), COX-2 and pro-inflammatory cytokines, and enhances the anti-inflammatory IL-10 in poly (ipurc)-stimulated microglia	Choi et al. (2017)
	Water extract of *A. officinarum*	Male NC/Nga mice	30, 100, and 300 mg/kg	The anti-inflammatory effect of *A. officinarum* water extract is related to its inhibitory effect on mitogen-activated protein kinase, nuclear factor kappa B, and signal transduction pathway 1	Song et al. (2021)
	Bioactive components of *A. officinarum*	RAW 264.7 mice; macrophages	0, 12.5, 25, and 50 mM	Galangin has an anti-inflammatory effect on endotoxin-activated macrophages by inhibiting the expression of ERK, NF-kB-p65, and pro-inflammatory genes	Li et al. (2021)
Antioxidant	Total flavonoids of *A. officinarum* (TFAO)	Male ICR mice	Determination of GSH-Px activity: 5,10,20 mg/L, TFAO 40 μL, determination of MDA content: 10,20,40 mg/L, TFAO 0.2 mL; erythrocyte oxidative hemolysis: 0.2 mL 0.5, 1.0, 2.0 mg/L TFAO	TFAO can effectively scavenge O^2-^, ·OH and DPPH·, and its ability of scavenging O^2-^ is stronger than that of the traditional antioxidant VC; It can significantly enhance the activity of GSH-Px in mouse liver and brain homogenate, effectively inhibiting the production of MDA, maintaining the integrity of cell membranes, inhibiting erythrocyte oxidative hemolysis induced by H_2_O_2_, and reducing tissue oxidative damage	Xia et al. (2009)
	Total flavonoids of *A. officinarum*	*Staphylococcus aureus, Escherichia coli, Bacillus subtilis, Pseudomonas aeruginosa, Candida albicans*	2.5 mL, purity over 98%	In terms of antioxidation, quercetin showed good antioxidant activity, while galangin had the lowest antioxidant activity. However, but the activity of galangin was similar to that of kaempferol and kaempferol in the ABTS radical scavenging test	Wang et al. (2017)
	Total flavonoids of *A. officinarum*		0.01, 0.025, 0.05, 0.1, 0.15, 0.20 mg/mL	The scavenging rate of total flavonoids of *A. officinarum* on DPPH radical increased with the increase in concentration. The scavenging rate was lower than that of Vc, with an IC50 of 0.05 mg/mL, which was equivalent to the IC50 of BHT	Shi et al. (2012)
	Total flavonoids of *A. officinarum*	Male ICR mice	Low, medium and high dose: 100, 100, 300, and 500 mg/kg	TFAO can significantly increase the activities of antioxidant enzymes (GSH-Px, SOD, CAT) and the content of GSH in lead-poisoned mice, improving lipid peroxidation and providing significant protection against lead poisoning-induced oxidative stress	Xia et al. (2013)
	Galangin	C57 male mice	25 mg/kg lasted until 4 weeks after operation	Galangin attenuates cardiac fibrosis induced by AB by reducing cardiac oxidative stress and inhibiting the transformation of cardiac fibroblasts into myofibroblasts	Yang et al. (2020)
	Galangin	Male KM mice; NRK-52E cells	Low, medium, and high doses: 100,200,400 mg/kg	Galangin can significantly inhibit the activation of NLRP3 inflammasomes and the release of IL-1β and IL-18 in NRK-52E cells	Lu et al. (2020)
	Galangin	Female Spraguee-Dawley rats; bilateral ovariectomy model	300 mg/(kg·d), last for 12 weeks	The ethanol extract of AOH can significantly reverse bone loss, in part by increasing bone formation and inhibiting bone resorption associated with its antioxidant effect	Su et al. (2016)
	*A. officinarum* oil	*Staphylococcus aureus, Escherichia coli, Bacillus subtilis, Saccharomyces cerevisiae*	0.02 g/mL	The peroxide value (POV) and acid value (AV) of peanut oil of *A. officinarum* volatile oil were lower	Huang et al. (2015)
	Different components of *A. officinarum* extract	HepG2 hepatoma cell line; HepG2 oxidative damage model induced by H_2_O_2_ in human hepatoma cell line	High, medium, and low doses: 300, 200 and 100 mg, administered continuously for 30 days	The diphenylheptane fraction of *A. officinarum* extract showed antioxidant-related activity *in vitro* and *in vivo*, followed by flavonoids	Lin et al. (2017)
Anti-liver injury	*A. officinarum*	Kunming mice, half male and half female; alcohol-induced acute alcoholic liver injury model in mice	Low, medium, and high dose: 1, 2, and 4 g/kg	*A. officinarum* may have a protective effect on alcoholic liver injury in mice by scavenging free radicals and providing antioxidant effect. However, its active components and specific mechanism need to be further studied	Zhou et al. (2012)
	Galangin	C57BL/6 mice; concanavalin A (ConA)-induced hepatitis model	25 mg/kg or 50 mg/kg	Galangin inhibits NF-κB and STAT1 signal transduction, resulting in a decrease in the expression and secretion of many inflammatory mediators	Luo et al. (2015)
Hypoglycemic	*A. officinarum*	ICR male mice	200 mg/kg	An 80% ethanol elution site can significantly reduce the blood glucose level of acute hyperglycemic mice	C et al. (2017)
	*A. officinarum* and its extract	Male New Zealand White Rabbit	4 g/kg	After oral administration of 3 and 4 g/kg *A officinarum* root powder for 4–8 h, the blood glucose level of normal rabbits decreased significantly	Akhtar et al. (2002)
	*A. officinarum* extract	Male Wistar rats; type 2 diabetic rats induced by nicotinamide/streptozotocin as model	100, 200, and 500 mg/kg for 28 days	The rhizome extract of *A. officinarum* exhibits antidiabetic effects in rats with type 2 diabetes	Heidari et al. (2022)
Hypolipidemic	Total flavonoids of *A.* officinarum (TFAO)	Male SD rats	Low, medium, and high doses: 100, 200, 200, and 300 mg	TFAO has significant effects on regulating blood lipids, antioxidation and protecting liver, and can regulate the expression of obesity-related factors, which may be the mechanism of its slimming and lipid-lowering effect	Fang et al. (2015)
Anticoagulant	*A. officinarum* and its main components	Wistar rat model of left carotid artery thrombosis	The water extract of *A. officinarum* is 10, 20 g/kg, and the ether extract of *A. officinarum* is 0.2 and 0.4 g/kg	The water extract of *A. officinarum* and the volatile oil of *A. officinarum* have obvious inhibitory effect on thrombosis in rats and have certain anticoagulant effect, which mainly participate in the endogenous coagulation system to improve the blood flow state	Xu et al. (1991)
Antibacterial	*A. officinarum* flavonoids	VVISA standard strain Mu50; methicillin resistant *Staphylococcus aureus* standard strains N315 and ATCC25293	0, 4, 8, and 16 μg/mL	Galangin can effectively inhibit the activity of murein hydrolase and the growth of VISA strain Mu50	Ouyang et al. (2018)
	Quercetin	*Pseudomonas aeruginosa* PAO1	125–256 μg/mL	Quercetin is an effective drug for inhibiting the formation of bacterial biofilm and virulence factors	Ouyang et al. (2016)
	Diarylheptanoid	*Candida albicans* (SC5314)	5 mg/mL	The chloroform extract of *A. officinarum* has the strongest antibacterial activity	Zhao et al. (2007)
	Effective components of volatile oil	16 strains of bacteria	Seven concentrations of 50, 25, 12.5, 6.25, 3.13, 1.56, and 0.78 μL/mL	The activity of *A. officinarum* volatile oil against the above-mentioned superficial dermatophytes is mainly by inhibiting their growth	Gui et al. (2005)
	*A. officinarum* extract	*Staphylococcus aureus, Pseudomonas aeruginosa, Candida albicans, Acinetobacter, E. coli*	Four concentrations of 0.5 g/ml, 0.25 g/mL, 0.125 g/mL, and 0.0625 g/mL	The extract of *A. officinarum* has good bacteriostatic effect on *Staphylococcus aureus*, but has no bacteriostatic effect on *Pseudomonas aeruginosa*, *Candida albicans*, *Acinetobacter* and *Escherichia coli*	Qin et al. (2015)
Improve memory ability	Different extracts of *A. officinarum*	Kunming mice; model of memory acquisition impairment induced by scopolamine in mice	3.33 mg/kg	Improve the ability of scavenging free radicals, reduce the levels of free radicals, and enhance the function of the central cholinergic nervous system	Liu et al. (2010a)
	Galangin	Kunming mice	Low and high dose 10 mg/kg, 20 mg/kg for 2 weeks, once a day	Galangin may play a role in delaying aging by increasing the activity of antioxidant enzymes and reducing the production of free radicals	Fu et al. (2012)
	Galangin	APP/PS1 double transgene mice; C57BL/6 mice	Low, medium and high dose 25 mg/kg, 50 mg/kg, 100 mg/kg	Galangin may improve learning and memory impairment in mice by regulating the Akt/MEF2D/Beclin-1 signaling pathway	Huang et al. (2022)
	*A. officinarum* extract	PC12 Cell	1, 10, 20, and 50 μg	The extract of *A. officinarum* could significantly reduce the leakage rate of intracellular lactate dehydrogenase, decrease the content of intracellular MDA and increase the activities of SOD and GSH-Px in a concentration-dependent manner	Zhai et al. (2014b)
	*A. officinarum* extract	Kunming mouse; model of memory consolidation disorder induced by sodium nitrite in mice	6.66, 3.33, 1.67 mg/kg (all in terms of crude drug quantity), once a day for 13 consecutive days	The mechanism may be related to improving the scavenging ability of free radicals and reducing the levels of free radicals	Liu et al. (2010b)
	*A. officinarum* extract	Kunming mice; model of memory acquisition impairment induced by scopolamine in mice	6.66, 3.33, 1.67 mg/kg (all in terms of crude drug quantity)	Both the water extract and ethanol extract of *A. officinarum* could significantly improve the histological changes in the hippocampus of mice with memory acquisition impairment	Zhao et al. (2010)
	*A. officinarum* extract	Kunming mouse; mouse model of acute cerebral ischemia; mouse model of memory acquisition impairment induced by berberine	6.66, 3.33, 1.67 mg/kg (all calculated by 3.33 mg/kg)	The water extract of *A. officinarum* can effectively reduce the brain water content and cerebral vascular permeability after acute cerebral ischemia	Chen (2012)
Anti-tumor	Galangin	Human hepatocellular carcinoma SMMC-7721	MTT method: 5.4, 10.8, 21.6, 43.2, 86.4 μg/mL; flow cytometry to analyze cell cycle/apoptosis: 10.8, 21.6, 43.2 μg/mL	Galangin may play a role in inducing apoptosis of the human hepatoma cell line SMMC-7721 through the PI3K/AKT signaling pathway	Liu et al. (2014)
	Galangin	Seven kinds of tumor cells	5, 10, 20, 40, 80, and 160 μmol/L	The inhibitory effect of galangin on different tumor cells was time-and concentration-dependent	Luo and Liu (2020)
	Galangin	Human osteosarcoma MG-63 cells	20, 40, 80, and 100 mM	Galangin can inhibit the proliferation and induce apoptosis of human osteosarcoma MG-63 cells, and its mechanism is related to the mitochondrial pathway	Song et al. (2012)
	Galangin	Cervical cancer SiHa cells	150, 125, 100, 75, 50, and 25 μg/mL	Galangin can induce apoptosis by increasing the transcription level of the apoptosis executive on factor caspase 3 and promoting the degradation of intracellular structural proteins	Abudula (2016)
	Galangin	Hepatoma cell	134, 87.3, and 79.8 μmol/L	Galangin induces apoptosis in HCC by activating the cysteine aspartate protease 8/t-Bid mitochondrial pathway	Zhang et al. (2012)
	Kaempferol	HCCLM3 and Huh7 cells	40, 80, and 120 μM	Kaempferol induces cell cycle arrest in HCC cells by regulating the ATM/CHEK2/KNL1 signaling pathway	Li et al. (2024)
	*A. officinarum* extract	MCF-7, LNCaP, and fibroblast cells	25, 50, 100, 200, and 400 μg/mL	*A. officinarum* extract induces apoptosis in two types of cancer cells	Kazemi et al. (2022)

### 5.1 Effects on gastrointestinal function

*A. officinarum* is an essential medicine for treating deficiency-cold of the spleen and stomach, as well as epigastric cold pain in traditional Chinese medicine. It is mainly used in the treatment of digestive tract diseases such as dyspepsia, acid reflux, and gastric ulcers. Wei (2019) used anhydrous ethanol and aspirin to induce two types of gastric ulcers models to study the effects of different extracts of *A. officinarum* on mice with gastric ulcers. The results showed that the aqueous extract of *A. officinarum* had a good anti-ulcer effect and decreased the ulcer index. It was inferred that the mechanism of the anti-ulcer effect of *A. officinarum* may be through inhibiting inflammatory factors, reducing gastrin (GAS), increasing cyclooxygenase-2 (COX-2), and prostaglandin E2 (PGE2), thereby enhancing the protective effect of the gastric mucosa and reducing gastric injury. Wang et al. (2011) studied the therapeutic effect of the volatile oil of *A. officinarum* on gastric ulcers. The results showed that the volatile oil of *A. officinarum* could reduce the gastric ulcer index and increase the ulcer inhibition rate in mice. *A. officinarum* reduces the levels of serum motilin (MOT) and substance P (SP), while increasing the levels of serum somatostatin (SS) and vasoactive intestinal peptide (VIP) in order to exert its anti-ulcer effect. In addition, the study found that the volatile oil of *A. officinarum* can increase the levels of serum nitric oxide (NO), expand the blood vessel walls, improve the microcirculation of the gastric mucosa, strengthen the mucosal barrier, scavenge oxygen free radicals, and protect the normal function of the gastric mucosa.

**FIGURE 4:**
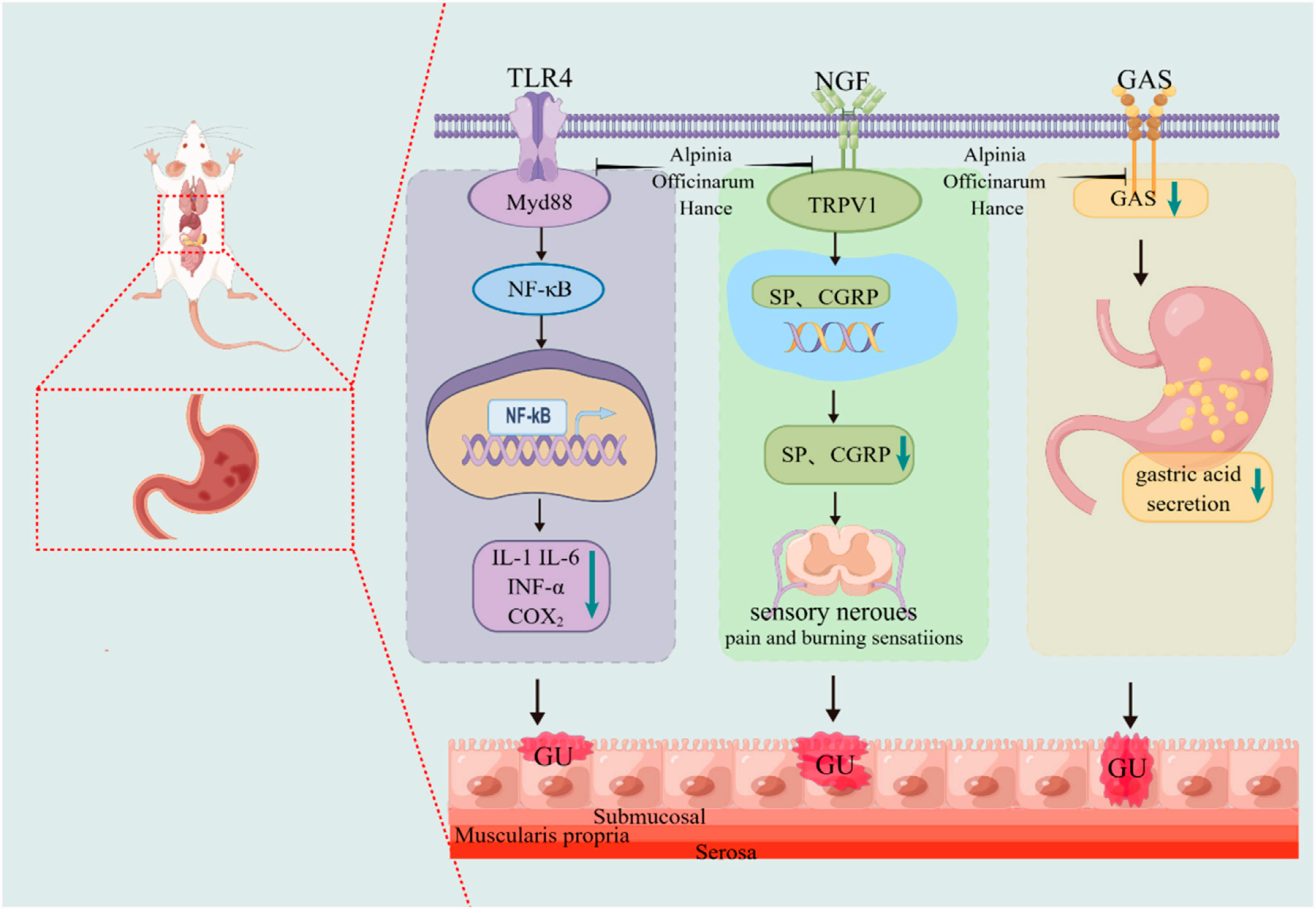
The mechanism of *A. officinarum* in improving gastrointestinal function. Toll-like receptor 4 (TLR4), nerve growth factor (NGF), calcitonin gene-related peptide (CGRP), gastric ulcer (GU).

*A. officinarum* has an obvious gastrointestinal spasmolytic effect, and its decoction can inhibit gastrointestinal propulsive movement. Gui et al. (2021) observed the effect of the total flavonoids of *A. officinarum* on the propulsive movement of the small intestine in normal rats using the charcoal powder method. The results showed that the total flavonoids of *A. officinarum* not only significantly inhibited the intestinal motility of normal rats, but also antagonized the hyperfunction of the small intestine induced by neostigmine. The mechanism may be that it affects the secretion and release of gastrointestinal hormones, such as somatostatin and vasoactive intestinal peptide, thus relaxing the smooth muscle. Cheng Yuan et al. (Cheng et al., 2015) studied the effects of various active components of *A. officinarum* on intestinal spasms induced by acetylcholine and on normal intestinal muscles in isolated rabbits. The results showed that the active components of *A. officinarum* extract could inhibit the spontaneous movement of intestinal muscles in a dose-dependent manner. Among these components, flavonoids and diphenylheptanes were the most prominent, and they were stronger than anisodamine. The mechanism of *A. officinarum* in improving gastrointestinal function is shown in Figure 4.

### 5.2 Analgesic and anti-inflammatory effect

**FIGURE 5:**
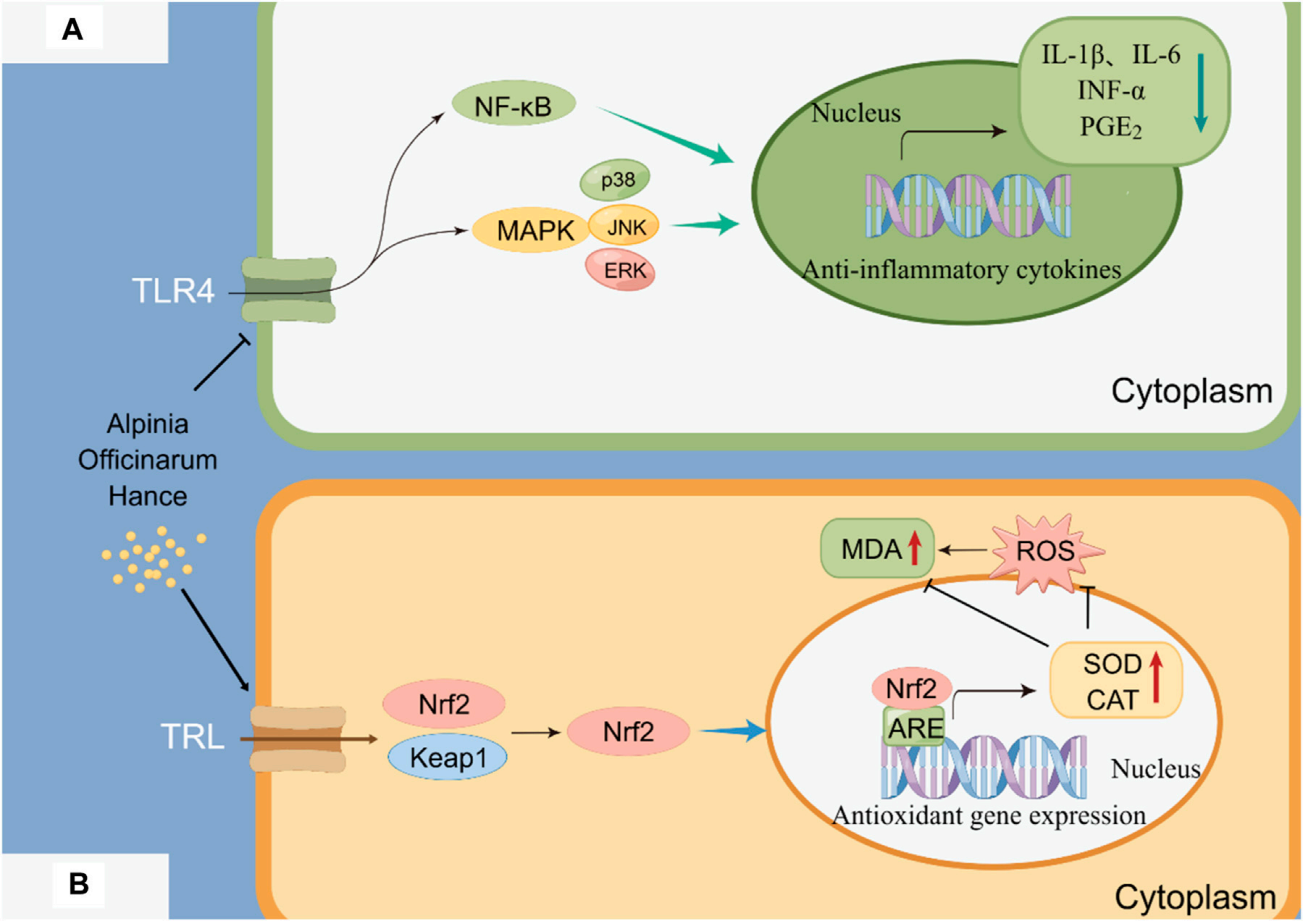
Anti-inflammatory and antioxidant mechanisms of *A. officinarum.* **(A)** Anti-inflammatory mechanism of total flavonoids of *A. officinarum.* **(B)** Antioxidant mechanism of extract of *A. officinarum*.

*A. officinarum* is hot and pungent, which has the effect of dispelling cold and relieving pain. As the use of non-steroidal anti-inflammatory drugs for long-term treatment of inflammation can cause obvious side effects, plants are constantly being developed as potential anti-inflammatory agents. Chen et al. (2009) used the carrageenan rat foot swelling model, the xylene mouse ear swelling model, and a capillary permeability experiment to study the anti-inflammatory effect of the total flavonoids extracted from *A. officinarum*. The mouse hot plate method and torsion test were used to observe the analgesic effect of the total flavonoids extracted from *A. officinarum*. The results showed that the total flavonoids extracted from *A. officinarum* had a significant inhibitory effect on acute inflammation models, such as toe swelling induced by carrageenan, auricle swelling induced by xylene, and an increase in celiac capillary permeability induced by acetic acid in mice. The total flavonoids of *A. officinarum* can inhibit pain induced by acetic acid and heat stimulation in mice. Liang et al. (2013) studied the therapeutic and analgesic effects of total flavonoids from *A. officinarum* (GLJ) on acetic acid-induced visceral hypersensitivity in rats with irritable bowel syndrome (IBS). The results showed that GLJ had a certain inhibitory effect on pain induced by heat stimulation, acetic acid, and formaldehyde in mice. Zha Wangjian et al. (Cha, 2015) found that galangin can inhibit airway inflammation and airway hyperresponsiveness to some extent in a mouse model of asthma. In addition, *A. officinarum* and its main compounds have anti-inflammatory effects on LPS-induced inflammation in RAW264.7 cells. This may be related to the inhibition of NF-κB activation. The anti-inflammatory mechanism of the total flavonoids of *A. officinarum* is shown in Figure 5A.

### 5.3 Antioxidant effect

An antioxidant is a type of active substance that can eliminate the inhibition of lipid peroxidation by free radicals. It can prevent the damage caused by lipid peroxidation to organisms. In a comparative study on the antioxidant activity of various components of *A. officinarum* extract, Lin et al. (2017) discovered that the diphenylheptanes exhibited antioxidant activity both *in vitro* and *in vivo*. Xia et al. (2009) found that the total flavonoids of *A. officinarum* can act as antioxidants by inhibiting reactive oxygen free radicals and decreasing the catalytic activity of metal ions *in vitro*. In the HepG2 oxidative damage model induced by H_2_O_2_, diphenylheptane in *A. officinarum* showed significant antioxidant activity. The extract of *A. officinarum* could potentially prevent oxidative stress damage by activating the Keap1/Nrf2/ARE signaling pathway. The antioxidant mechanism of the extract of *A. officinarum* is shown in Figure 5B.

### 5.4 Antibacterial effect

The *in vitro* antibacterial experiment conducted by Zhao et al. (2007) showed that the chloroform and ethyl acetate extracts of *A. officinarum* exhibited anti-*Candida albican*s activity. The chloroform extract of *A. officinarum*, at a concentration of 20 mg/mL, demonstrated strong activity. Qin et al. (2015) showed that both the alcohol extract and water extract of *A. officinarum* had a good inhibitory effect on methicillin-resistant *Staphylococcus aureus*, but had no significant inhibitory effect on *Pseudomonas aeruginosa*, *Candida albicans*, *Acinetobacter*, or *Escherichia coli*. Flavonoids are the most important antibacterial components of *A. officinarum*. Ouyang et al. (2018) studied the impact of galangin on the antibacterial activity against vancomycin-intermediate *S. aureus*. The study results showed that galangin had significant inhibitory activity against ATCC25293, N315, and Mu50, with a minimum inhibitory concentration (MIC) of 32 mg/L. The results of further studies showed that galangin inhibited the growth of bacteria by inhibiting the activity of cell wall hydrolase. At the same time, the effect of quercetin on *P. aeruginosa* PAO1 was also studied (Ouyang et al., 2016). The results showed that 16 mg/L of quercetin could significantly inhibit the biofilm formation, the quorum sensing system, and independent factors of *P. aeruginosa*. This suggests that quercetin may have the potential to treat biofilm-associated infections.

### 5.5 Improve memory ability

Alzheimer’s disease (AD) is a chronic degenerative disease of the central nervous system in middle-aged and elderly individuals. Its main clinical manifestation is cognitive dysfunction. Huang Liping (Huang et al., 2022) has shown that galangin can improve learning and memory impairment in APP/PS1 mice. It may inhibit the activity of acetylcholinesterase (AChE) in the brain through the cholinergic pathway, increasing the level of ACh and improving learning and memory function. On the other hand, it may play a role in protecting hippocampal neurons by regulating the Akt/MEF2D/Beclin-1 signaling pathway and clearing abnormal proteins in hippocampal neurons through autophagy and chaperone-mediated autophagy (CMA). This can reduce the deposition of amyloid-β (Aβ) and the formation of tau protein. It can be concluded that galangin may improve the learning and memory impairment of APP/PS1 mice by regulating the Akt/MEF2D/Beclin-1 signaling pathway. In the PC12 cell injury model stimulated by H_2_O_2_, *A. officinarum* extract can significantly reduce the lactate dehydrogenase leakage rate, decrease the content of MDA, and increase the activities of SOD and GSH-Px (Zhai et al., 2014b).

### 5.6 Anti-tumor effect

The anti-tumor mechanism of *A. officinarum* can be reflected in regulating the cell cycle, inducing tumor cell apoptosis and autophagy, inhibiting tumor cell migration and invasion, and reversing drug resistance in tumors. Luo and Liu (2020) found that galangin has a broad-spectrum anti-tumor effect. Its inhibitory effect on different tumor cells varies and depends on time and concentration. Galangin can strongly inhibit the genotoxicity of chemical toxic substances *in vivo* and *in vitro*, making it a potential preventive drug for cancer. Zhang et al. (2012) found that *A. officinarum* can induce apoptosis by activating mitochondrial apoptosis, caspases, and causing changes in the levels of Bcl-2 in various liver cancer cell lines. Additionally, kaempferol derived from *A. officinarum* has the ability to induce apoptosis in HCCLM3 and Huh7 cells by controlling the ATM/CHEK2/KNL1 signaling pathway.

### 5.7 Other functions

In addition to the above pharmacological effects, *A. officinarum* has anti-liver injury, hypoglycemic, hypolipidemic, and anticoagulant effects. Zhou et al. (2012) showed that *A. officinarum* can protect the function of hepatocytes in mice after an acute alcoholic liver injury. The results showed that *A. officinarum* could significantly reduce the concentrations of alanine aminotransferase (ALT) and aspartate transaminase (AST) in the serum of mice after injury, indicating that *A. officinarum* has a certain hepatoprotective effect. Its pharmacological mechanism may be to protect liver cells by scavenging free radicals and reducing the degree of damage caused by alcohol. Akhtar et al. (2002) showed that the extract of *A. officinarum* has a significant hypoglycemic effect. In the hypoglycemic experiment on normal male New Zealand rabbits, oral *A. officinarum* powder at a dose of 3 g/kg significantly reduced blood glucose levels. The methanol and water extracts showed even more pronounced hypoglycemic effects. When the oral dose was increased to 4 g/kg, there was a significant decrease in blood glucose levels of rabbits after 8 h. However, *A. officinarum* powder and its extract had no effect on rabbits with diabetes induced by alloxan. Therefore, its hypoglycemic effect may be achieved by promoting insulin secretion from the pancreas in the body. Obese patients are often accompanied by abnormal fat metabolism, which can lead to high blood total cholesterol (TC) and/or triglyceride (TG) levels. Fang et al. (2015) showed that middle and high doses of total flavonoids from *A. officinarum* play a significant role in controlling body mass, fat accumulation, and cholesterol metabolism, as well as reducing the levels of serum leptin and plasma neuropeptide Y in nutritionally obese rats with hyperlipidemia. A study (Luo et al., 2015) has shown that galangin has an obvious inhibitory effect on thrombosis in rats, demonstrating a certain anticoagulant effect. The potential mechanism may be to improve the blood flow state of rats by participating in the endogenous coagulation system.

## 6 Conclusion and prospection

*A. officinarum* is an important traditional Chinese medicine for both medicine and food. Using modern research methods, the pharmacological effects of its active compounds have been clearly described, and the mechanisms of anti-gastric ulcer, inhibition of gastrointestinal motility, antioxidant effect, antibacterial, anti-inflammatory, and analgesia have been gradually clarified. The treatment of traditional digestive tract diseases has been expanded to a certain extent, broadening its scope of clinical application. So far, 337 compounds have been isolated from *A. officinarum.* Among them, galangin is a very important active compound extracted from *A. officinarum*. The pharmacological effects of galangin are very extensive. However, most pharmacological effects are currently only verified in cell and animal models, and there is a lack of clinical study data to support them. In addition, the mechanism of pharmacological action of galangin is not fully understood. Most studies are limited to the pharmacodynamic level or a few specific targets or pathways, and are unable to elucidate the general mechanism of action or the connection between the various targets and pathways. In the future, based on existing research, network pharmacology, bioinformatics, and multi-omics analysis can be used to comprehensively and deeply analyze the molecular mechanisms, genes, and signaling pathways of galangin. Further studies are needed to explore the extracts of *A. officinarum* for any potential toxicities, side effects, and contraindications. With the continuous discovery of the structure of the active components of *A. officinarum* and the in-depth study of its pharmacological activity, its pharmacodynamic mechanism is gradually becoming clear. The research scope of the pharmacological activity of *A. officinarum* has been continuously expanded by the vast number of scientific research works, and its medicinal value will be further developed and applied.

## Abbreviations

AD, Alzheimer’s disease; AUC, area under the curve; AchE, acetylcholinesterase; ALT, alanine aminotransferase; AST, aspartate transaminase; C_max_, peak concentration; COX-2, cyclooxygenase-2; CMA, chaperone-mediated autophagy; DIC, disseminated intravascular coagulation; iNOS, inducible nitric oxide synthase; GG-1, galangin-3-O-β-D-glucuronic acid; GG-2, galangin-7-O-β-D-glucuronic acid; IBS, irritable bowel syndrome; Ka, absorption rate constants; MDA, malondialdehyde; MIC, minimum inhibitory concentration; MOT, motilin; NO, nitric oxide; GAS, gastrin; Papp, apparent absorption coefficients; PGE2, prostaglandin E2; SOD, superoxide dismutase; SP, substance P; SS, serum somatostatin; t_peak_, peak time; t_1/2ke_, elimination half-life; t_1/2ka_, absorption half-life; TC, total cholesterol; TG, triglycerides; VIP, vasoactive intestinal peptide; TLR4, toll-like receptor 4; NGF, nerve growth factor; CGRP, calcitonin gene-related peptide; GU, gastric ulcer.
